# The Behavior of Phenolic Compounds from Apples during Simulated Gastrointestinal Digestion with Focus on Chlorogenic Acid

**DOI:** 10.3390/foods13050693

**Published:** 2024-02-24

**Authors:** Lidija Jakobek, Kristina Pöc, Matea Valenteković, Petra Matić

**Affiliations:** Faculty of Food Technology Osijek, Josip Juraj Strossmayer University of Osijek, Franje Kuhača 18, 31 000 Osijek, Croatia; kpoc@ptfos.hr (K.P.); mvalentekovic@ptfos.hr (M.V.); petra.matic@ptfos.hr (P.M.)

**Keywords:** neochlorogenic acid, cryptochlorogenic acid, digestive tract, bioeffects, bioaccessibility

## Abstract

The fate of phenolic compounds during digestion is important for their bioactive effects in the digestive tract. The aim was to study the various phenolic compounds occurring in the peel and flesh of apples in in vitro simulated gastrointestinal digestion, focusing on the behavior of chlorogenic acids. Additionally, the behavior of individual chlorogenic acids (chlorogenic, neochlorogenic, and cryptochlorogenic) was studied in models of simulated salivary, gastric, and intestinal fluid electrolyte solutions (SSF, SGF, SIF). At the end of the intestinal phase of the digestion of peel and flesh, the amount of recovered dihydrochalcones and flavonols increased or was similar to the amount in the gastric phase, which showed their stability. Anthocyanins and flavan-3-ols decreased, which suggests their biotransformation. Chlorogenic acid isomerized into neochlorogenic and cryptochlorogenic acid: chlorogenic acid from the peel into 22% and 41% of the isomers in the salivary and intestinal phases, respectively; chlorogenic acid from the flesh into 12% of the isomers in the intestinal phase. Similarly, chlorogenic acid isomerized in model solutions (20% and 26% of the isomers in SSF and SIF, respectively). Neochlorogenic and cryptochlorogenic acid isomerized in SSF and SIF into other two forms. They were all stable in SGF. For bioactive effects in the digestive tract, the biotransformation of chlorogenic acids should be considered.

## 1. Introduction

Apples are used worldwide in the diet and are, therefore, an important source of phenolic compounds. They are not the fruit with the highest amounts of phenolic compounds, but they are a fruit available through the whole year and liked by consumers. There are several potential benefits of phenolic compounds from consuming apples, especially for the digestive system, which is the system that first comes into contact with polyphenols, and we highlight here potential beneficial effects in the stomach and intestines. Concerning the stomach, some studies showed the potential beneficial effects of apple polyphenols in gastric ulcer healing [1,2], or in stomach infections caused by *Helicobacter pylori* [3,4,5]. In addition, they can alleviate changes in the gastric mucosa caused by consuming nonsteroidal anti-inflammatory drugs (NSAID) [6]. Likewise, apple polyphenols showed the potential for beneficial effects in the small intestine and the colon. They can strengthen the gut barrier and be helpful in alleviating the condition called “leaky gut” [7,8]. Phenolic compounds from apples have the potential for lowering glucose absorption by inhibiting the digestive enzymes important in the digestion of starch, which is helpful in alleviating hyperglycemia [9,10,11]. Studies showed their positive influence in preserving the gut microbiota balance [12,13,14], and in colon cancer [15,16]. Due to all of these mentioned potentially positive bio-effects in the digestive tract, and the widespread use of apples, the forms and amounts of phenolic compounds from apples released in digestion is important.

Phenolic acids are one of the most dominant classes of phenolic compounds in apples [17,18]. Though they vary somewhat depending on the apple variety, phenolic acids occupy about 40% of the total phenolic compounds of the flesh, and about 10% of the total phenolic compounds of the peel [17]. The most prevalent individual phenolic acid in apples is chlorogenic acid, as variously quantified in the whole fruit of some varieties of traditional and exotic apples [19], in the pulp of some rare apples from Italy [20], in many various apple varieties [17], or in the flesh of ancient apple cultivars [21]. In apples grown organically or conventionally, chlorogenic acid was again the predominant compound [22]. According to the mentioned studies, chlorogenic acid is an important phenolic compound in apples with its high percentage within the total phenolic compound amount. Therefore, its role in the potential beneficial effects of apples in the digestive system may be important. It is known that chlorogenic acid can isomerize into cryptochlorogenic and chlorogenic acid in the environmental conditions of the digestive tract. However, more studies are necessary to understand this process, and to understand the fate of isomers of chlorogenic acids and their potential degradation in the environments of the different phases of digestion. Some studies showed the potential of the isomer cryptochlorogenic acid to attenuate inflammatory symptoms [23,24] and to inhibit oxidative stress [23]. Anti-inflammatory effects were shown for the isomer neochlorogenic acid as well [25]. Thus, due to the potential beneficial effects of chlorogenic acid isomers, their behavior in different phases of the digestive system should be better known.

The aim of this study was to conduct the in vitro simulated gastrointestinal digestion of the peel and flesh of apples to investigate the fate of all phenolic compounds, but in particular the fate of chlorogenic acid in digestion. Moreover, the aim was to investigate the behavior of chlorogenic acid and its isomers (cryptochlorogenic and neochlorogenic acid) in the salivary, gastric, and intestinal electrolyte solutions to see the behavior of each of the individual acids under the environmental conditions of the digestion phases.

## 2. Materials and Methods

### 2.1. Standards, Chemicals, and Solutions

Authentic standards of phenolic compounds were obtained from Sigma-Aldrich (St. Louis, MO, USA) ((+)-catechin hydrate, (−)-epicatechin, quercetin dihydrate, quercetin-3-glucoside, *p*-coumaric acid, chlorogenic acid, neochlorogenic acid, and cryptochlorogenic acid) and from Extrasynthese (Genay, France) (procyanidins B1 and B2, quercetin-3-galactoside, quercetin-3-rhamnoside, phloretin, phloretin-2′-*O*-glucoside, and cyanidin-3-galactoside chloride). Other chemicals such as potassium chloride, potassium dihydrogen phosphate, sodium hydrogen carbonate, calcium chloride, and magnesium chloride were obtained from Gram mol d.o.o. (Zagreb, Croatia), ammonium carbonate from Kemika d.d. (Zagreb, Croatia), and sodium chloride from Carlo Erba Reagents GmbH (Val de Reuil, France). Orto-phosphoric acid, 85% HPLC grade and methanol HPLC grade were purchased from Fluka (Buchs, Switzerland) and J.T. Baker (Gliwice, Poland), respectively. Furthermore, alpha amylase (A6255, >1000 U/mg), pepsin (P7000, 632 U/mg), pancreatin (P7545, 8 USP), and bile salt (B 8756, microbiology grade) were from Sigma-Aldrich (St. Louis, MO, USA).

The stock solutions of electrolytes were prepared in the following concentrations: (KCl, KH_2_PO_4_, and (NH_4_)_2_CO_3_ (0.5 M), MgCl_2_ (0.15 M), CaCl_2_ (0.3 M), NaHCO_3_ (1 M), NaCl (2 M)). Simulated salivary fluid electrolyte solution (SSF), simulated gastric fluid electrolyte solution (SGF), and simulated intestinal fluid electrolyte solution (SIF) were prepared from stock solutions of electrolytes with the final concentrations: SSF (18.875 mmol/L KCl, 4.625 mmol/L KH_2_PO_4_, 17 mmol/L NaHCO_3_, 0.056 mmol/L MgCl_2_, and 0.06 mmol/L (NH_4_)_2_CO_3_, with pH measured at neutral 7); SGF (8.625 mmol/L KCl, 1.125 mmol/L KH_2_PO_4_, 31.25 mmol/L NaHCO_3_, 0.15 mmol/L MgCl_2_, 0.625 mmol/L (NH_4_)_2_CO_3_, and 59 mmol/L NaCl, with pH adjusted to 3 with 1 M HCl); and SIF (8.5 mmol/L KCl, 1 mmol/L KH_2_PO_4_, 106.25 mmol/L NaHCO_3_, 0.4125 mmol/L MgCl_2_, and 48 mmol/L NaCl, with pH adjusted to 7 with 1 M HCl). Enzyme solutions were prepared daily (α-amylase 1500 U/mL in SSF, pepsin 31,660.61 mg/L in SGF, pancreatin 8000 mg/L in SIF) and preincubated at 37 °C before use. Bile salts were prepared in SIF (25,000 mg/L).

### 2.2. Apple Samples and the Extraction of Phenolic Compounds

A total of 1 kg of mature red peel apples (Pink Lady) with yellowish flesh were purchased in a local supermarket (located in town Osijek, Croatia) and peeled. After peeling the apples, the residue of flesh was removed from the peel with a knife and the remaining apple peels were pooled together. The core was removed from the flesh of the apples and the remaining flesh was cut into smaller pieces. The peel and flesh were weighed, dried in a food dehydrator machine, weighed again, and homogenized in a coffee grinder. The homogenized samples were stored at −18 °C and used for experiments the same week that they were prepared.

To extract phenolic compounds from the peel and flesh of the apples, dried samples of peel (around 0.03 g) and flesh (around 0.08 g) were weighed into plastic tubes; 1.5 mL of 80% methanol in water was added into the tubes, the solutions were homogenized in a vortex (PV-1, Grant Instruments, Cambridgeshire, UK), extracted in an ultrasonic bath (Bandelin Sonorex RK 100, Berlin, Germany) for 15 min, and centrifuged (Eppendorf Minispin, Eppendorf, Hamburg, Germany) for 10 min at 10,000 rpm. Extracts were removed from residues by pipetting and residues were extracted again with the same procedure using 0.5 mL of 80% methanol. After combining the two extracts from the peel, and combining the two extracts from the flesh, the resulting extracts were filtered (0.20 μm PTFE syringe filter) and analyzed with reversed-phase high-performance liquid chromatography (RP-HPLC) (Agilent technology, Santa Clara, CA, USA) explained further in the text.

The remaining phenolic compounds in the residue were extracted with the help of enzymes. Distilled water (1.05 mL), bile salt (60 μL), pancreatin (30 μL), and pepsin (15 μL) were added to the peel and flesh residues remaining after the first chemical extraction. The solutions were vortexed and incubated in a dry block thermostat (Bio TDB-100, Biosan, Riga, Latvia) at 37 °C for 2 h with vortexing repeated every 10 min. To stop the reaction, the solutions were put in an ice bath (5 °C, 5 min), centrifuged (5 min at 10,000 rpm), and again put in an ice bath (5 °C, 5 min). Extracts were removed by pipetting, then filtered (0.20 μm PTFE syringe filter) and analyzed with RP-HPLC.

To obtain the amounts of phenolic compounds in the peel and flesh of apples before the digestion, the amounts from chemical and enzymatic-assisted extractions were added.

### 2.3. Simulated Digestion

Simulated digestion was conducted according to a procedure already described in [26] with some modifications. For the simulated digestion in the mouth, peel (0.08 g) and flesh (0.03 g) were weighed in two plastic tubes. Then 175 μL of SSF, 49 μL of distilled water, 25 μL of α-amylase, and 1.25 μL of CaCl_2_ were added into each tube. The solutions were vortexed for 30 s and incubated in a dry block thermostat at 37 °C for 5 min. The tubes were put in an ice bath (5 °C, 5 min), centrifuged (10,000 rpm, 5 min), and again put in an ice bath until analysis.

For the simulated digestion in the stomach, peel (0.08 g) and flesh (0.03 g) were weighed in two plastic tubes. The simulated digestion in the mouth was conducted again according to the procedure already described. Then the simulated digestion in the stomach was continued by adding 375 μL of SGF, 15 μL of H_2_O, 0.25 μL of CaCl_2_, 10 μL of HCl (1 M), and 100 μL of pepsin to the solutions after the oral digestion. The solutions were vortexed and incubated in a dry block thermostat (2 h, 37 °C). The vortexing was conducted for 5 min each. Tubes were put in an ice bath (5 °C, 5 min), centrifuged (10,000 rpm, 5 min), and again put in an ice bath until analysis.

For the simulated digestion in the small intestine, peel (0.08 g) and flesh (0.03 g) were weighed in two plastic tubes. The simulated digestion in the mouth and stomach was conducted according to the procedures already described. The simulated digestion in the small intestine was continued by adding 550 μL of SIF, 180.5 μL of H_2_O, 2 μL of CaCl_2_ (0.3 M), 7.5 μL of NaOH (1M), 250 μL of pancreatin, and 10 μL of bile salt to the solutions after oral and gastric digestion. The solutions were vortexed and incubated in a dry block thermostat (37 °C, 2 h) with vortexing every 5 min. After 2 h, the tubes were put in an ice bath (5 °C, 5 min), centrifuged (10,000 rpm, 5 min), and again put in an ice bath until analysis.

For analysis, solutions after oral, gastric, and intestinal digestion were pipetted from the residue, filtered (0.20 μm PTFE syringe filter), and analyzed with RP-HPLC. If the solution after oral and gastric digestion of the apple flesh was too dense to pipette the solution, SSF or SGF were added into solutions, solutions were vortexed, centrifuged, and then separated from the residue by pipetting.

The recovery was calculated as:

(1) $$\mathrm{recovery}\;\left(\%\right)=\frac{\gamma_{\mathrm{digestion}\;\mathrm{phase}}\;\left(\mathrm{mg}/\mathrm{kg}\right)}{\gamma_{\mathrm{before}\;\mathrm{digestion}}\;\left(\mathrm{mg}/\mathrm{kg}\right)}\;100$$

where γ_digestion phase_ is the concentration of a phenolic compound after a particular digestion phase (mg/kg fresh weight (FW)) and γ_before digestion_ is the concentration of that phenolic compound in fruit before digestion determined with chemical and enzyme-assisted extraction (mg/kg FW).

### 2.4. Reversed-Phase High-Performance Liquid Chromatography (RP-HPLC)

Samples were analyzed with reversed-phase chromatography using a Poroshell 120 EC C-18 column (4.6 × 100 mm, 2.7 μm), a Poroshell 120 EC-C18 4.6 mm guard-column, and an instrument 1260 Infinity II, with a quaternary pump, a PDA detector, and a vialsampler (Agilent technology, Santa Clara, CA, USA). The separation was achieved by using 0.1% H_3_PO_4_ (mobile phase A), and 100% methanol (mobile phase B) with a gradient 0 min 5% B, 5 min 25% B, 14 min 34% B, 25 min 37% B, 30 min 40% B, 34 min 49% B, 35 min 50% B, 58 min 51% B, 60 min 55% B, 62 min 80% B, 65 min 80% B, 67 min 5% B, and 72 min 5% B [20]. The flow was 0.5 mL/min, and each sample was injected in 10 μL. To identify phenolic compounds, samples were spiked with authentic standards. In addition, phenolic compounds were identified by comparing UV/Vis spectra and retention time with those of authentic standards. The quantification was conducted using calibration curves. Quercetin-3-xyloside, quercetin derivative, and *p*-coumaroylquinic acid were identified with the help of the literature and quantified using quercetin and *p*-coumaric acid calibration curves.

### 2.5. Degradation of Chlorogenic Acids in Simulated Digestion Electrolyte Solutions (SSF, SGF, SIF)

The degradation of chlorogenic acids that can be found in apples (chlorogenic, neochlorogenic, and cryptochlorogenic acids) was studied in model solutions by preparing each chlorogenic acid separately in SSF, SGF, and SIF, by analyzing them immediately and after 4 h incubation with RP-HPLC. In short, for the analysis before the degradation in simulated SSF, SGF, and SIF, each chlorogenic acid was separately prepared in SSF, SGF, and SIF, and immediately analyzed on the RP-HPLC. That would be the amount of chlorogenic acids before the degradation. Next, for the analysis after the degradation, each chlorogenic acid was separately prepared in SSF, SGF and SIF, incubated 4 h at 37 °C, and analyzed in the RP-HPLC. All reaction solutions were prepared by pipetting 1470 μL of appropriate simulated solution and 30 μL of stock solutions (1000 mg/L) of chlorogenic acids, to get the final concentration to be 20 mg/L. Compounds found after 4 h are expressed as percentages of the height of the peak in comparison to the height of the peak of the parent compound at the beginning of the incubation.

### 2.6. Statistical Analysis

The extraction of phenolic compounds with the chemical and enzyme-assisted extraction was repeated in two parallel measurements, and each were analyzed twice with the RP-HPLC (n = 4). The experiment of simulated digestion was conducted in two parallel measurements and analyzed once with the RP-HPLC (n = 2). The experiment of the degradation of chlorogenic acids was conducted once and analyzed once with RP-HPLC. The data were analyzed with a post-hoc Tukey test using Minitab software (21.4.) (Minitab LLC., State College, PA, USA).

## 3. Results

### 3.1. Phenolic Compounds in Apples

The chromatograms of the extracts of the peel and flesh of apples before digestion, labeled with the identified phenolic compounds, are shown in Figure S1. Phenolic compounds from the peel were identified as compounds from five different classes usually found in apples: anthocyanin (cyanidin-3-galactoside), flavan-3-ols (monomeric (+)-catechin, (−)-epicatechin, and dimeric procyanidins B1 and B2), dihydrochalcones (phloretin-2′-glucoside), phenolic acids (chlorogenic acid), and flavonols (quercetin-3-galactoside, quercetin-3-glucoside, an unknown quercetin derivative, quercetin-3-xyloside, and quercetin-3-rhamnoside). The flesh of the apples contained the same classes of phenolic compounds as in the peel, except for a difference in phenolic acids and a lack of any anthocyanins. The phenolic acids in the flesh were identified as chlorogenic acid, cryptochlorogenic acid, an unknown phenolic acid, and *p*-coumaroylquinic acid. The same phenolic compounds were identified in apples in earlier studies [17,18,19,20,21,22].

**Figure 1:**
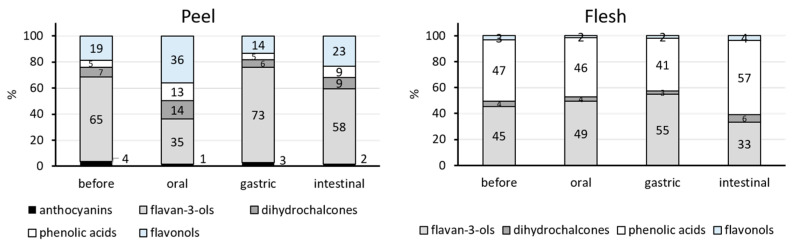
Percentage distribution of phenolic classes from peel and flesh before the digestion and after simulated digestion. Percentages are calculated according to the total amount of phenolic compounds.

Table 1 and Table 2, respectively, show the amounts of identified phenolic compounds in the peel and the flesh before digestion, and, likewise, Figure 1 shows their percentage distribution. One of the main differences between the flesh and peel was the total amount of phenolic compounds. The peel had higher amounts of phenolic compounds (2163 mg/kg FW) than the flesh (644 mg/kg FW). The distribution of phenolic classes was also different. In the peel, flavan-3-ols were represented with the highest amount (1401 mg/kg FW), followed by flavonols (403 mg/kg FW), dihydrochalcones (160 mg/kg FW), phenolic acids (116 mg/kg FW), and anthocyanins (84 mg/kg FW) (Table 1). As the two main classes in the peel, flavan-3-ols and flavonols occupied the highest percentages, 65% and 19% of the total amount, respectively (Figure 1). An earlier study also reported that the peel is characterized by higher concentrations of flavan-3-ols and flavonols, which occupied 40% and 36% of the peel polyphenols, respectively [17]. The flesh had the highest amount of phenolic acids (305 mg/kg FW), followed by flavan-3-ols (293 mg/kg FW), dihydrochalcones (27 mg/kg FW), and flavonols (19 mg/kg FW) (Table 2). Two main classes, phenolic acids and flavan-3-ols, occupied 47% and 45% of the total amount, respectively (Figure 1). The higher amount of phenolic acids and flavan-3-ols in the flesh was mentioned in the earlier study [17]. They occupied 53% and 39% of the total amount, respectively [17], similar to this study. Wojdyło et al. [18] also reported that flavan-3-ols and phenolic acids were the dominant classes in whole apples.

### 3.2. Phenolic Compounds in Simulated Digestion

Figure S2 shows chromatograms of the peel and flesh of apples after the three phases of simulated digestion, in the mouth, stomach, and small intestine. There were some differences in the identified compounds, in comparison to the identification before the digestion. Only chlorogenic acid was identified in the peel before digestion, while additional phenolic acids were identified after the oral and intestinal phases, specifically, neochlorogenic and cryptochlorogenic acid. After digestion of the flesh, a new phenolic acid was identified in the intestinal phase (neochlorogenic acid). Moreover, the unknown quercetin derivative was not detected after digestion of the flesh. The differences in the phenolic acids during digestion were already reported in an earlier study [27].

The released and recovered amounts of total and individual phenolic compounds in the three phases of digestion are shown in Table 1 and Table 2 and the percentage distributions of phenolic classes in Figure 1. Phenolic compounds from the peel were released in the oral phase (Table 1) in the total amount of 501 mg/kg FW. Their amount significantly increased in the gastric phase to 1421 mg/kg FW (*p* < 0.05), and then decreased in the intestinal phase, but not significantly, to 1211 mg/kg FW. Flavan-3-ols (35% to 73%) and flavonols (14% to 36%) occupied the highest percentages in the total recovered phenolic compounds from the peel in all three phases of digestion (Figure 1). The total amounts of phenolic compounds from the flesh released in the oral and gastric phases were similar (495 and 485 mg/kg FW, respectively), and decreased in the intestinal phase to 346 mg/kg FW. Those changes were not statistically significant. Flavan-3-ols (33% to 55%) and phenolic acids (41% to 57%) occupied the highest percentages in all three phases (Figure 1).

During the simulated digestion, phenolic compounds gradually released in the mouth and stomach, after which their amounts decreased in the intestine, but not significantly. However, the dominant classes of polyphenols after each phase of digestion remained the same as before the digestion—flavan-3-ols and flavonols dominated after the digestion of the peel, and flavan-3-ols and phenolic acids after the digestion of the flesh. In an earlier study, after the duodenal phase of digestion of whole apples or just the flesh of apples, phenolic acids dominated in the total released phenolic compounds [28]. After the digestion of the peel, the dominant class in the duodenal phase was flavonols [28].

#### Phenolic Classes in the Simulated Digestion

Anthocyanins. Anthocyanins (Table 1) were released in the oral phase, their amount increased in the gastric phase, and then decreased in the intestinal phase (*p* < 0.05). The reason for their variable recovered amounts is the pH of the gastrointestinal environment. In the oral phase, the pH was higher (pH 7), it decreased in the gastric phase (pH 3), and again increased in the intestinal phase (pH 7). The amounts of released anthocyanins were higher at the low pH value of the gastric phase where they were present in the flavylium cation form. By passing into the small intestine to a higher pH, they biotransformed into the carbionol pseudo-base form, which is colorless [29]. The biotransformed forms of anthocyanins were the reason for their lower measured amounts in the intestinal phase.

Flavan-3-ols. The amounts of recovered total flavan-3-ols from the peel (Table 1) significantly increased from the oral to the gastric phase, and then significantly decreased in the intestinal phase (*p* < 0.05). Individual flavan-3-ols showed the same behavior. The exception was (+)-catechin whose amount was similar in all three phases of digestion. Total flavan-3-ols from flesh (Table 2) were present in similar amounts in the oral and gastric phases, and their amount significantly decreased in the intestinal phase (*p* < 0.05). The amount of dimeric procyanidins followed the same trend: their amount was similar in the oral and gastric phase, and then decreased or they were not identified in the intestinal phase. However, the amount of released monomeric (+)-catechin and (−)-epicatechin was similar in all phases of digestion. The pH of different phases of digestion could play a significant role in the release of flavan-3-ols, in the mentioned differences, and in the decrease in the intestinal phase. It can be suggested that flavan-3-ols can degrade into unknown compounds when passing from the gastric phase with its lower pH to the small intestine with its higher pH, since their total measured amounts significantly decreased. Some earlier studies also suggested the degradation of flavan-3-ols in the intestinal phase [27,30]. If dimeric procyanidins degraded into monomeric units, the amount of monomeric flavan-3-ols would have been increased. However, the amount of monomeric (+)-catechin and (−)-epicatechin was similar in all phases of digestion or decreased in the intestine, which suggests that dimeric procyanidins did not degrade into monomeric units in this study.

Earlier studies showed various results. In our earlier studies [31,32], we did not identify flavan-3-ols in the intestinal phase of digestion. The reason for that could be the use of fresh fruit in our earlier studies, and therefore, the lower initial amount of flavan-3-ols in the digestion, which together with their degradation affected the loss in the intestinal phase. Similarly, when using fresh apples, Bouayed et al. [27] did not identify catechin, epicatechin, and procyanidins B1 and B2 in the intestinal phase. For this study, we used the dry apple material, which had higher initial amounts of flavan-3-ols and enabled us to identify flavan-3-ols in the intestinal phase, despite their decreased amount. In in-vitro-simulated digestion of freeze-dried apple peel, which is also a dry material, procyanidin B1 and catechin were found in the intestinal digestion similar to this study, and its amount increased from the gastric to intestinal digestion, while epicatechin derivatives were not found [33]. Flavan-3-ols were also identified in the intestinal phase in a low amount after the in-vitro-simulated digestion of some apple varieties (dried material), similar to our study [28]. Moreover, the in-vitro-simulated digestion of apples (dry material) resulted in identifiable procyanidins, which even increased from the gastric to the intestinal phase [34]. Opposite to our study, in the study of Fernández-Jalao et al. [30] after the in vitro dynamic gastrointestinal digestion of apples (dried, powdered sample), procyanidin B1, catechin, and procyanidin B2 were not found in the intestinal phase, but they did identify epicatechin in a decreased amount. Studies on human subjects offer more precise results on the fate of phenolic compounds during digestion. In a study on humans, in which subjects consumed apple juice, catechin and dimeric procyanidins were not found in their ileal fluid, while epicatechin was [35], similar to this study. Oligomeric procyanidins were also present in the ileal fluid [35]. This suggests a stability of oligomeric procyanidins in the human small intestine, while dimeric procyanidins might be cleaved into monomers and possibly absorbed [35].

Flavan-3-ols represented 35% to 73% (peel) or 33% to 55% (flesh) of the phenolic compounds in different phases of digestion (Figure 1).

Dihydrochalcones: Dihydrochalcone phloretin-2′-glucoside from the peel was recovered in similar amounts in the oral and gastric phases, and its amount increased in the intestinal phase (*p* < 0.05) (Table 1). The amount of recovered phloretin-2′-glucoside from the flesh was similar in all phases of digestion (*p* < 0.05) (Table 2). It can be suggested that phloretin-2′-glucoside was stable across the different pH values of the phases of digestion, which is consistent with earlier in vitro studies [27,28,34]. Indeed, in the studies of Tenore et al. [34] and Fernández-Jalao et al. [30], the amounts of phloretin-2′-glucoside from the peel and flesh of apples, or from whole apples, increased from oral to gastric and intestinal digestion, which established its stability. After human subjects consumed apple juice [35], phloretin-2′-xyloglucoside was detected in the ileal fluid, which again supported the stability of some dihydrochalcones at the higher pH of the intestine. However, phloretin-2′-glucoside was not identified, though its metabolite phloretin-2-glucuronide was [35]. Several suggestions concerning the fate of phloretin-2′-glucoside were provided in [35]. It might be hydrolyzed into aglycon phloretin and glucose. Phloretin might be absorbed, after which it reaches the liver where glucuronidation takes place. Phloretin-2-glucuronide can reach the small intestine through the enterohepatic cycle, or the glucuronidation can be the result of the enterocytes of the small intestine [35]. Accordingly, the study of ileal fluid offered additional views on the fate of dihydrochalcones in the small intestine. 

The percentage of dihydrochalcones was 6–14% (peel) or 3–6% (flesh) of the total compounds found in different phases of digestion.

Phenolic acids: The amount of total phenolic acids from the peel released in the oral and gastric phase was similar. However, it increased in the intestinal phase (*p* < 0.05) (Table 1). Even though this could point to the stability of phenolic acids in the digestion, there were some visible differences at the level of individual phenolic acids. Firstly, only chlorogenic acid could be detected in the peel before the digestion, and at the end of the gastric phase (Table 1). Additionally, neochlorogenic and cryptochlorogenic acid were identified after digestion in the oral and intestinal phases. The appearance of those additional acids could be the result of isomerization. Chlorogenic acid can isomerize into neochlorogenic and cryptochlorogenic acid at the higher pH of the oral and intestinal phases. It can be suggested that, due to the isomerization, a part of chlorogenic acid biotransformed into neochlorogenic and cryptochlorogenic acid at the higher pH of the oral and intestinal phases, while at the lower pH of the gastric phase, chlorogenic acid was stable.

The amounts of total released phenolic acids from the flesh were similar in all phases (*p* < 0.05) (Table 2). Some individual phenolic acids followed the same trend, namely, chlorogenic acid and *p*-coumaroylquinic acid. However, the amount of cryptochlorogenic acid increased in the intestine (*p* < 0.05). Again, chlorogenic acid may have isomerized at the higher pH of the intestine since the isomer neochlorogenic acid was identified, and the amount of cryptochlorogenic acid increased. 

The isomerization of chlorogenic acid into cryptochlorogenic and neochlorogenic acid in the intestine was confirmed in an earlier study [27]. However, in another in vitro study, the amount of chlorogenic acid was almost not affected by the three phases of digestion [30]. In the study on humans, when subjects with ileostomy consumed apple juice, native chlorogenic acid and cryptochlorogenic acid, together with the isomer neochlorogenic acid, were found in the ileal fluid. The authors also suggested the isomerization of chlorogenic acid in the human small intestine [35], similar to this study. 

Phenolic acids contributed to 5–13% (peel) and 41–57% (flesh) of the total phenolic compounds found in different phases of the digestion (Figure 1).

Flavonols: The total flavonols from the peel were released in the oral phase and then recovered in the gastric phase in similar amounts, while their amount increased passing through the intestinal phase (*p* < 0.05) (Table 1). At the individual level, all the individual flavonols showed the same behavior except for quercetin-3-galactoside whose amount was similar in all phases (Table 1). The amount of flavonols was much lower in the flesh, but it is still possible to see that their total recovered amount followed a similar trend—the amount increased through all three phases but not statistically significantly (Table 2). Similarly, the amount of individual flavonols from flesh increased in the intestinal phase, or their amount was similar in all phases (Table 2). The results suggest the stability of flavonols in all phases of digestion, at a low and higher pH. This is in accordance with earlier studies [27,33] in which the stability of flavonols was reported, likewise without identification of quercetin aglycon, which usually points to the hydrolysis of quercetin glycosides. In the earlier study [34], a flavonol from apples (quercetin-3-rutinoside) was recovered from the oral to the gastric phase in increased amounts, but its content decreased after the intestinal digestion. Similarly, Fernández-Jalao et al. [30] reported the stability of flavonols in gastric digestion while the amount of flavonols decreased at the end of the intestinal phase. They suggested the possibility of the hydrolysis of flavonols in the stomach and small intestine since the amount of aglycon quercetin increased. We did not find aglycon quercetin, which suggests that there was no hydrolysis of flavonols in this study. In the different phases of digestion, the percentage of flavonols was 14–36% (peel) or 2–4% (flesh) (Figure 1).

**Figure 2:**
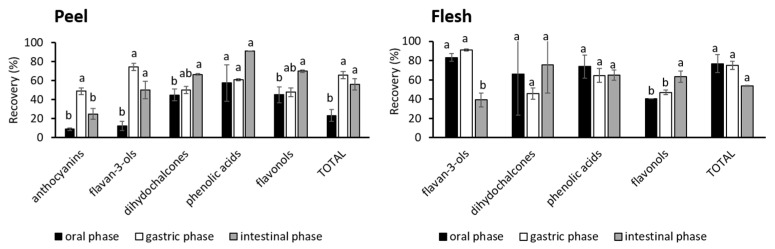
Recovery of phenolic classes from peel and flesh after oral, gastric, and intestinal phases of digestion. The values with different letters are significantly different according to the post-hoc Tukey test (*p* < 0.05), with letters a to b ordered from highest to lowest.

Figure 2 shows the percentage recovery of phenolic classes from the peel and flesh in all phases of digestion. The recovery of anthocyanins and flavan-3-ols increased from the oral to the gastric phase, and then decreased in the intestinal phase, although not always by a statistically significant amount. The decrease was due to their degradation/biotransformation already mentioned. The stability of dihydrochalcones and flavonols is visible since their recovery gradually increased, though not always significantly. The recovery of phenolic acids was similar in all phases.

### 3.3. The Degradation of Chlorogenic Acids from Apples during the Digestion

**Figure 3:**
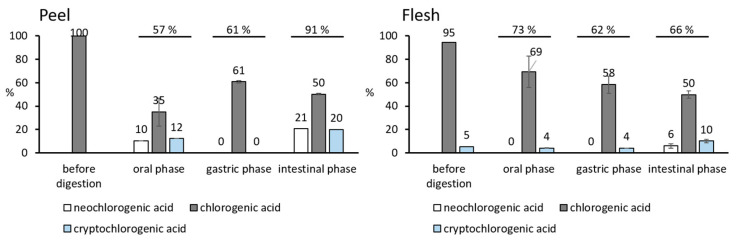
Percentages of neochlorogenic, chlorogenic, and cryptochlorogenic acid before digestion, and in the three phases of the digestion. Percentages are calculated as percentages of the amount found at the end of a specific digestion phase (mg/kg fresh weight fw) according to the total amount before digestion (mg/kg fresh weight fw).

The percentage distribution of different forms (isomers) of chlorogenic acids in the three phases of digestion is shown in Figure 3. Out of 100% of the chlorogenic acid in the peel of apples before the digestion, only 35% was released in the oral phase at a higher pH, while 10 and 12% were found in the form of isomers as neochlorogenic and cryptochlorogenic acid, respectively. Altogether, it seems that 57% of the chlorogenic acid was released in the oral phase in the form of the parent compound or isomers thereof. Furthermore, 61% was found in the gastric phase in the form of the parent compound as chlorogenic acid. It can be suggested that all forms found in the oral phase (57%) isomerized back into the form of chlorogenic acid at the lower pH of the gastric phase (61%), and some additional amount of chlorogenic acid might have been liberated from the peel. In the intestinal phase, chlorogenic acid was found in the parent form (50%) and in the form of two isomers (21% and 20% neochlorogenic and cryptochlorogenic acid, respectively). Once again, chlorogenic acid showed some isomerization at the higher pH. The total percentage of parent compound and isomers (91%) could point to the additional release of chlorogenic acid passing from the gastric (61%) to the intestinal phase (91%).

Out of 95% and 5% of the native chlorogenic and cryptochlorogenic acid found in the flesh of apples before the digestion, respectively (Figure 3), only 69% and 4% were released in the oral phase. In the further gastric phase, the percentage of recovered chlorogenic acid decreased to 58%, while it remained the same for cryptochlorogenic acid (4%). The isomerization can be seen in the further intestinal phase where 50% of chlorogenic acid as a parent compound was found, together with the isomers neochlorogenic acid (6%) and cryptochlorogenic acid (10%). Neochlorogenic acid was identified only at the end of the intestinal phase (6%), and the percentage of cryptochlorogenic acid increased from the gastric (4%) to the end of the intestinal phase (10%), which suggests the isomerization of chlorogenic acid in the intestine at the higher pH. The result was 12% of the created isomers (6% neochlorogenic, 6% cryptochlorogenic acid). Altogether, 66% of the chlorogenic acid and isomers was recovered in the intestinal phase.

### 3.4. The Degradation of Chlorogenic Acids in Simulated Salivary, Gastric, and Intestinal Electrolyte Solutions

**Figure 4:**
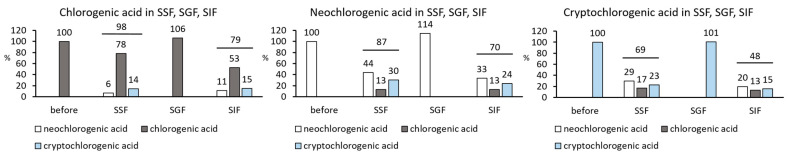
The isomerization of chlorogenic, neochlorogenic, and cryptochlorogenic acid in simulated salivary fluid (SSF) electrolyte solution, simulated gastric fluid (SGF) electrolyte solution, and simulated intestinal fluid (SIF) electrolyte solution, after a 4 h incubation at 37 °C.

To additionally study the biotransformation of chlorogenic acid, we studied its stability/degradation in simulated electrolyte solutions for salivary, gastric, and intestinal digestion (SSF, SGF, and SIF) (Figure 4). The incubation was studied for 4 h, which is the total time of the gastric and intestinal phases together. After 4 h, at pH 7 for SSF, chlorogenic acid isomerized into neochlorogenic (6%) and cryptochlorogenic acid (14%). Altogether, 98% of the chlorogenic acid in the form of all three acids was found in SSF. It can be suggested that chlorogenic acid isomerized but it did not degrade in SSF. In the SGF, at a lower pH only native chlorogenic acid was identified (106%). At a higher pH of the SIF, chlorogenic acid isomerized again, into 11% of the neochlorogenic acid and 15% of the cryptochlorogenic acid, while the percentage of parent chlorogenic acid was 53%. Altogether, 79% of the chlorogenic acids were found in SIF, which leads us to the suggestion that some degradation of chlorogenic acid into unknown products might have occurred in the SIF.

Neochlorogenic acid also showed isomerization (Figure 4). It isomerized into chlorogenic acid (13% in SSF and SIF) and cryptochlorogenic acid (30% and 24% in SSF and SIF, respectively) at the higher pH of SSF and SIF. It did not isomerize at the lower pH of the SGF. Totals of 87% and 70% of the initial amount were recovered in SSF and SIF respectively, in the form of parent neochlorogenic acid and two isomers, which suggests some degradation into unknown products at the higher pH of SSF and SIF.

Similarly, cryptochlorogenic acid showed isomerization. It isomerized in SSF and SIF into neochlorogenic acid (29% and 20% in SSF and SIF, respectively) and chlorogenic acid (17% and 13% in SSF and SIF, respectively). Only 69% and 48% of the initial amount was recovered in SSF and SIF, respectively, in the form of the parent compound and isomers, which again suggests the degradation of cryptochlorogenic acid into unknown products at a higher pH of the SSF and SIF. There was no isomerization of cryptochlorogenic acid in SGF at the lower pH.

Individual examination of the degradation of each of the three chlorogenic acids confirmed the isomerization at the higher pH of the mouth and intestine. The stability of all forms of chlorogenic acids at the lower pH of the stomach was shown.

## 4. Discussion

The major classes of phenolic compounds in the peel before and after all three phases of digestion (mouth, stomach, and intestine) were flavan-3-ols (35% to 73%) and flavonols (14% to 36%). In the case of apple flesh, the dominant classes were phenolic acids (41% to 57%) and flavan-3-ols (33% to 55%). Even though the peel has higher amounts of phenolic compounds (in our study 3.4 times more (2163 mg/kg FW) than flesh (644 mg/kg FW)), the flesh takes up the biggest part of the weight of the whole apple, and provides the major contribution of phenolic compounds when consuming the whole apple. For this reason, the classes of phenolic compounds found in the flesh might be very important for the beneficial effects of whole apples. Consequently, phenolic acids from the flesh might be important in the bioactivities of whole apples.

Chlorogenic acid as a major phenolic acid from apples (38.9% in the polyphenols of flesh), can be found in different forms in different phases of digestion. In the mouth, it can be in the form of parent chlorogenic acid, but it can also isomerize into neochlorogenic and cryptochlorogenic acid. The isomerization in the mouth was found for the chlorogenic acid from peel. Among the 57% of all forms released from the peel in the oral phase, 22% were in the form of isomers. Isomerization in the oral phase was not seen for the chlorogenic acid from apple flesh. Accordingly, for potential beneficial effects of apples in the mouth, all three forms might be important even though phenolic compounds stay in the mouth for a short period. Earlier studies did investigate the effects of various phenolic compounds in the mouth. Studies showed that phenolic compounds from Chinese gallnut extract [36] or beverages rich in phenolics [37] showed antibacterial activity [36] or reduced the number of detectable bacteria [37] on the enamel surface. In the stomach, chlorogenic acid appears only in its native form, as chlorogenic acid. It can be suggested that the beneficial effects in the stomach might be connected to the native form of chlorogenic acid. There are many studies supporting the potential beneficial effects of various phenolic compounds in the stomach. Phenolic compounds can inhibit the propagation of lipid peroxidation in the stomach and as a consequence decrease the amount of oxidized lipids in the bloodstream [38,39,40]. Phenolic compounds from various sources or from apples can also help in healing the gastric ulcers [2,41,42] or in being helpful in infections caused by *Helicobacter pylori* [5]. When considering such effects of apples, and more specifically the effects of chlorogenic acid from apples in the stomach, the effects of parent, native chlorogenic acid might be considered. For the beneficial effects in the small intestine, the effects of chlorogenic acid as well as its isomers neochlorogenic and cryptochlorogenic acid should be considered. Namely, out of 91% of all chlorogenic acids from the peel released in the intestinal phase, and out of 66% of chlorogenic acids from the flesh released in the intestinal phase, 41% (peel) and 16% (flesh) were in the form of isomers. The higher pH value of the salivary and intestinal phases is the probable cause of the observed isomerization. Various studies showed the potential of phenolic compounds from apples to be helpful in the small intestine through maintaining the barrier function of the small intestine [6,8] or in helping with problems caused by chronic administration of nonsteroidal anti-inflammatory drugs [6]. The potential effects of chlorogenic acid from apples in the small intestine should be considered together with the effects of its isomers.

The isomerization of chlorogenic acid was confirmed in the model solutions of SSF and SIF electrolyte solutions. Out of 100% of the initial chlorogenic acid, 98% was found after 4 h of incubation in the SSF. In those 98%, 20% was present in the form of isomers and the other was the parent, chlorogenic acid. Out of 100% of the initial chlorogenic acid, 79% was found after 4 h of incubation in SIF. In those 79%, 26% was present in the form of isomers and the other part was the parent, chlorogenic acid. The isomerization of chlorogenic acid at the higher pH into neochlorogenic acid and cryptochlorogenic acid is not the only biotransformation that occurs during the digestion. As shown, the other two forms can also biotransform. Neochlorogenic acid isomerized into chlorogenic and cryptochlorogenic acid (43% and 37% of isomers in SSF and SIF, respectively). Cryptochlorogenic acid isomerized into chlorogenic and neochlorogenic acid (46% and 33% of isomers in SSF and SIF, respectively). It can be suggested that those three processes of isomerization occur at the same time, transferring one form into another.

Isomers have been studied for beneficial effects: cryptochlorogenic acid for anti-inflammatory activity and antioxidative effects [23,24], and neochlorogenic acid for anti-inflammatory activity [25]. The bioactivities of cryptochlorogenic acid and neochlorogenic acid should be considered when explaining the beneficial effects of apples in the digestive system due to their relatively high amount (in the mouth up to 22% of the total chlorogenic acid amount before digestion of the peel; in the small intestine 41% and 12% of the total chlorogenic acid amount before digestion of the peel or flesh, respectively).

## 5. Conclusions

The main phenolic classes from apples behaved differently during digestion. At the end of the intestinal phase, the amount of recovered dihydrochalcones and flavonols increased or was similar to the amount in the gastric phase, which showed their stability. The amounts of anthocyanins and flavan-3-ols decreased, which suggests their biotransformation during digestion. Chlorogenic acid isomerized into neochlorogenic and cryptochlorogenic acid during digestion in the oral and intestinal phases: chlorogenic acid from the peel into 22% and 41% of the isomers in the oral and intestinal phases, respectively; chlorogenic acid from the flesh into 12% of the isomers in the intestinal phase. Similarly, chlorogenic acid showed isomerization in model solutions that simulate the electrolytes and the pH of salivary and intestinal fluids (simulated salivary and intestinal fluid electrolyte solutions, SSF and SIF, respectively). After 4 h of incubation, chlorogenic acid isomerized into 20% and 26% of the isomers in SSF and SIF, respectively. The isomerization of parent chlorogenic acid from apples shown during the digestion in the mouth and small intestine was confirmed in the simulated solutions. Moreover, the two isomers of chlorogenic acid, namely neochlorogenic and cryptochlorogenic acid, also isomerized in simulated salivary and intestinal fluid electrolyte solutions into the other identified forms (neochlorogenic into 43% and 37% of the isomers in SSF and SIF, respectively; cryptochlorogenic acid into 46% and 33% of the isomers in SSF and SIF, respectively). The isomers should be considered when studying the beneficial effects of apples in the mouth or small intestine. Since isomerization occurs in time, the kinetics of the release of chlorogenic acid and its biotransformation into isomers might also be studied. The effect of the matrix of an apple could also be significant for isomerization and beneficial effects.

## Figures and Tables

**Table 1:** Amounts of phenolic compounds from an apple peel before digestion and after phases of simulated digestion (mg/kg fresh weight (fw)).

	Before Digestion	Oral Phase	Gastric Phase	Intestinal Phase
Anthocyanins				
cyanidin-3-galactoside	83.5 ± 2.6 ^a^	7.4 ± 1.0 ^d^	40.7 ± 3.0 ^b^	20.8 ± 4.6 ^c^
total	83.5 ± 2.6 ^a^	7.4 ± 1.0 ^d^	40.7 ± 3.0 ^b^	20.8 ± 4.6 ^c^
Flavan-3-ols				
procyanidin B1	134.2 ± 5.5 ^a^	18.9 ± 10.1 ^c^	142.9 ± 4.4 ^a^	70.8 ± 12.2 ^b^
(+)-catechin	163.6 ± 14.0 ^a^	20.2 ± 7.0 ^b^	56.8 ± 8.5 ^b^	46.1 ± 19.4 ^b^
procyanidin B2	456.6 ± 12.0 ^a^	46.0 ± 27.1 ^c^	484.3 ± 23.1 ^a^	323.8 ± 68.3 ^b^
(−)-epicatechin	646.2 ± 15.3 ^a^	88.9 ± 22.6 ^d^	353.7 ± 16.7 ^b^	257.5 ± 26.8 ^c^
total	1400.6 ± 35.1 ^a^	174.0 ± 66.7 ^d^	1037.7 ± 52.8 ^b^	698.2 ± 126.8 ^c^
Dihydrochalcones				
phloretin-2′-glucoside	160.1 ± 8.0 ^a^	71.5 ± 10.0 ^c^	80.1 ± 5.9 ^c^	106.1 ± 1.4 ^b^
total	160.1 ± 8.0 ^a^	71.5 ± 10.0 ^c^	80.1 ± 5.9 ^c^	106.1 ± 1.4 ^b^
Phenolic acids				
neochlorogenic acid	nd	11.8 ± 4.1 ^b^	nd	23.9 ± 0.3 ^a^
chlorogenic acid	115.7 ± 4.1 ^a^	40.4 ± 13.8 ^c^	70.4 ± 1.3 ^b^	58.2 ± 0.5 ^b,c^
cryptochlorogenic acid	nd	14.2 ± 4.0 ^b^	nd	23.0 ± 0.3 ^a^
total	115.7 ± 4.1 ^a^	66.4 ± 21.9 ^b^	70.4 ± 1.3 ^b^	105.1 ± 0.1 ^a^
Flavonols				
quercetin-3-galactoside	70.4 ± 3.9 ^a^	32.9 ± 9.7 ^b^	36.4 ± 3.8 ^b^	46.8 ± 1.0 ^b^
quercetin-3-glucoside	42.6 ± 1.3 ^a^	19.1 ± 2.8 ^c^	19.3 ± 1.7 ^c^	33.7 ± 1.7 ^b^
quercetin derivative *	55.5 ± 1.1 ^a^	20.3 ± 3.1 ^c^	21.7 ± 1.9 ^c^	34.8 ± 0.4 ^b^
quercetin-3-xyloside *	95.5 ± 1.7 ^a^	39.5 ± 6.6 ^c^	41.2 ± 3.6 ^c^	63.2 ± 0.9 ^b^
quercetin-3-rhamnoside	138.9 ± 1.8 ^a^	69.8 ± 11.2 ^c^	73.9 ± 7.5 ^c^	102.5 ± 5.9 ^b^
total	402.9 ± 9.8 ^a^	181.6 ± 33.5 ^c^	192.5 ± 18.6 ^c^	281.0 ± 5.7 ^b^
TOTAL	2162.8 ± 50.3 ^a^	500.9 ± 133.1 ^c^	1421.4 ± 81.5 ^b^	1211.2 ± 124.4 ^b^

Amounts before the digestion are mean values of two parallel experiments measured two times in HPLC (n = 4); amounts in the gastric and intestinal digestion are mean values of two parallel experiments each measured once in HPLC (n = 2). The values in rows with different letters are significantly different according to the post-hoc Tukey test (*p* < 0.05), with letters a to d ordered from highest to lowest. * Tentatively identified compounds.

**Table 2:** Amounts of phenolic compounds from apple flesh before digestion and after phases of simulated digestion (mg/kg fresh weight (fw)).

	Before Digestion	Oral Phase	Gastric Phase	Intestinal Phase
Flavan-3-ols				
procyanidin B1	38.9 ± 6.4 ^b^	57.3 ± 0.4 ^a^	65.4 ± 1.6 ^a^	Nd
(+)-catechin	43.6 ± 5.2 ^a^	19.6 ± 2.1 ^b^	18.3 ± 0.0 ^b^	17.2 ± 0.0 ^b^
procyanidin B2	121.7 ± 19.5 ^a^	112.1 ± 6.1 ^a^	126.1 ± 1.6 ^a^	53.1 ± 18.0 ^b^
(−)-epicatechin	88.9 ± 14.1 ^a^	55.1 ± 3.9 ^b^	56.7 ± 0.3 ^b^	44.6 ± 3.4 ^b^
total	293.1 ± 45.2 ^a^	244.1 ± 11.7 ^a^	266.5 ± 3.5 ^a^	114.9 ± 21.4 ^b^
Dihydrochalcones				
phloretin-2′-glucoside	26.8 ± 5.3 ^a^	17.7 ± 11.4 ^a^	12.3 ± 1.6 ^a^	20.3 ± 7.8 ^a^
total	26.8 ± 5.3 ^a^	17.7 ± 11.4 ^a^	12.3 ± 1.6 ^a^	20.3 ± 7.8 ^a^
Phenolic acids				
neochlorogenic acid	nd	Nd	nd	16.0 ± 4.9 ^a^
chlorogenic acid	250.4 ± 37.8 ^a^	183.7 ± 35.1 ^a,b^	154.5 ± 19.5 ^b^	132.1 ± 7.8 ^b^
cryprochlorogenic acid	14.5 ± 1.8 ^b^	10.9 ± 0.5 ^b^	10.5 ± 0.2 ^b^	26.9 ± 4.0 ^a^
unknown phenolic acid *	29.4 ± 0.9 ^a^	23.1 ± 0.6 ^b^	24.8 ± 1.9 ^b^	18.5 ± 0.7 ^c^
*p*-coumaroylquinic acid *	11.0 ± 1.9 ^a^	7.4 ± 0.6 ^a,b^	7.1 ± 0.4 ^a,b^	4.7 ± 0.1 ^b^
total	305.3 ± 40.7 ^a^	225.1 ± 36.8 ^a,b^	196.9 ± 22.1 ^b^	198.2 ± 16.0 ^b^
Flavonols				
quercetin-3-galactoside	0.1 ± 0.0 ^b^	0.2 ± 0.0 ^a^	0.2 ± 0.0 ^a,b^	0.1 ± 0.0 ^b^
quercetin-3-glucoside	7.6 ± 0.3 ^a^	3.6 ± 0.2 ^c^	4.5 ± 0.3 ^c^	6.2 ± 0.5 ^b^
quercetin derivative	4.6 ± 0.1 ^a^	Nd	nd	Nd
quercetin-3-xyloside	4.8 ± 0.2 ^a^	2.4 ± 0.1 ^c^	3.0 ± 0.2 ^c^	4.2 ± 0.3 ^b^
quercetin-3-rhamnoside	2.1 ± 0.5 ^a^	1.5 ± 0.4 ^a^	1.4 ± 0.0 ^a^	1.7 ± 0.3 ^a^
total	19.2 ± 1.2 ^a^	7.7± 0.0 ^c^	9.1 ± 0.5 ^b,c^	12.2 ± 1.1 ^b^
TOTAL	644.4 ± 92.3 ^a^	494.6 ± 59.9 ^a,b^	484.8 ± 27.7 ^a,b^	345.6 ± 3.6 ^b^

Amounts before the digestion are mean values of two parallel experiments measured two times in HPLC (n = 4); amounts in the gastric and intestinal digestion are mean values of two parallel experiments each measured once in HPLC (n = 2). The values in rows with different letters are significantly different according to the post-hoc Tukey test (*p* < 0.05), with letters a to c ordered from highest to lowest. * Tentatively identified compounds.

## Data Availability

The data presented in this study are available in the Supplementary Material, Figures S1 and S2. Further inquiries can be directed to the corresponding author.

## Supplementary Materials

The following supporting information can be downloaded at: https://www.mdpi.com/article/10.3390/foods13050693/s1, Figure S1: Chromatograms of extracts after the chemical extraction (before digestion)—apple peel and flesh, scanned at 280, 320, 360, and 510 nm. Peak identification: 1—procyanidin B1, 2—neochlorogenic acid, 3—(+)-catechin, 4—procyandin B2, 5—chlorogenic acid, 6—cryptochlorogenic acid, 7—(−)-epicatechin, 8—unknown phenolic acid, 9—cyanidin-3-galactoside, 10—*p*-coumaroylquinic acid, 11—quercetin-3-galactoside, 12—quercetin-3-glucoside, 13—quercetin derivative, 14—phloretin-2-glucoside, 15—quercetin-3-xyloside, 16—quercetin-3-rhamnoside; Figure S2: Chromatograms after the simulated digestion in the mouth, stomach, and small intestine—apple peel and flesh. Peak identification: 1—procyanidin B1, 2—neochlorogenic acid, 3—(+)-catechin, 4—procyandin B2, 5—chlorogenic acid, 6—cryptochlorogenic acid, 7—(−)-epicatechin, 8—unknown phenolic acid, 9—cyanidin-3-galactoside, 10—*p*-coumaroylquinic acid, 11—quercetin-3-galactoside, 12—quercetin-3-glucoside, 13—quercetin derivative, 14—phloretin-2-glucoside, 15—quercetin-3-xyloside, 16—quercetin-3-rhamnoside.
