# Supplementary material for: The Behavior of Phenolic Compounds from Apples during Simulated Gastrointestinal Digestion with Focus on Chlorogenic Acid
Source: Foods. 2024 Feb 24;13(5):693. doi: 10.3390/foods13050693 (PMC10931301; doi:10.3390/foods13050693)
Supplement: Supplementary file 1 [file foods-13-00693-s001.zip › foods-2815698-supplementary.pdf]

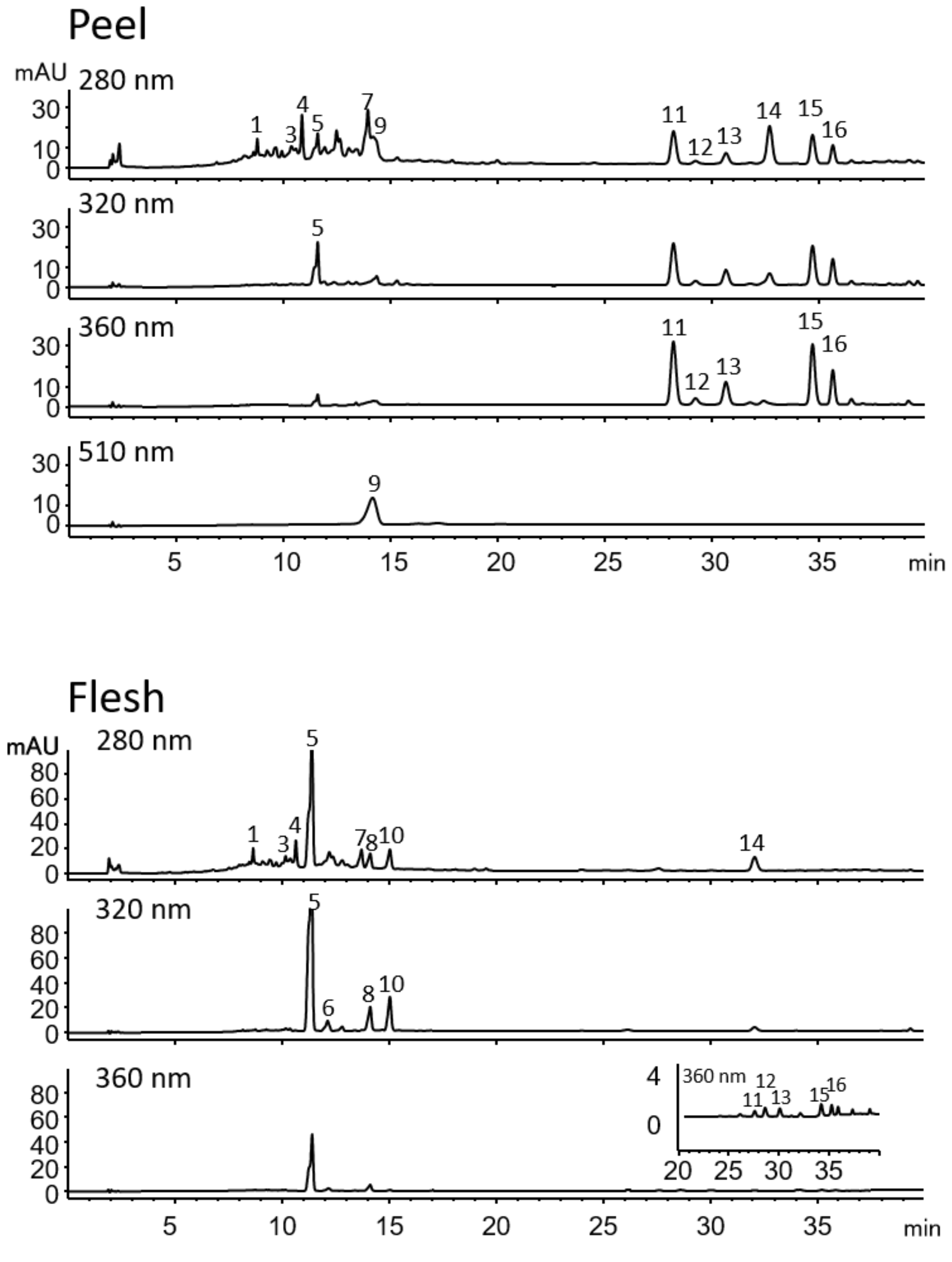

Figure S1. Chromatograms of extracts after the chemical extraction (before digestion) – apple peel and flesh, scanned at 280, 320, 360 and 510 nm. Peak identification: 1 – procyanidin B1, 2 – neochlorogenic acid, 3 – (+)-catechin, 4 – procyandin B2, 5 – chlorogenic acid, 6 – cryptochlorogenic acid, 7 – (-)-epicatechin, 8 – unknown phenolic acid, 9 – cyanidin-3-galactoside, 10 – *p*-coumaroylquinic acid, 11 – quercetin-3-galactoside, 12 – quercetin-3-glucoside, 13 – quercetin derivative, 14 – phloretin-2′-glucoside, 15 – quercetin-3-xyloside, 16 – quercetin-3-rhamnoside.

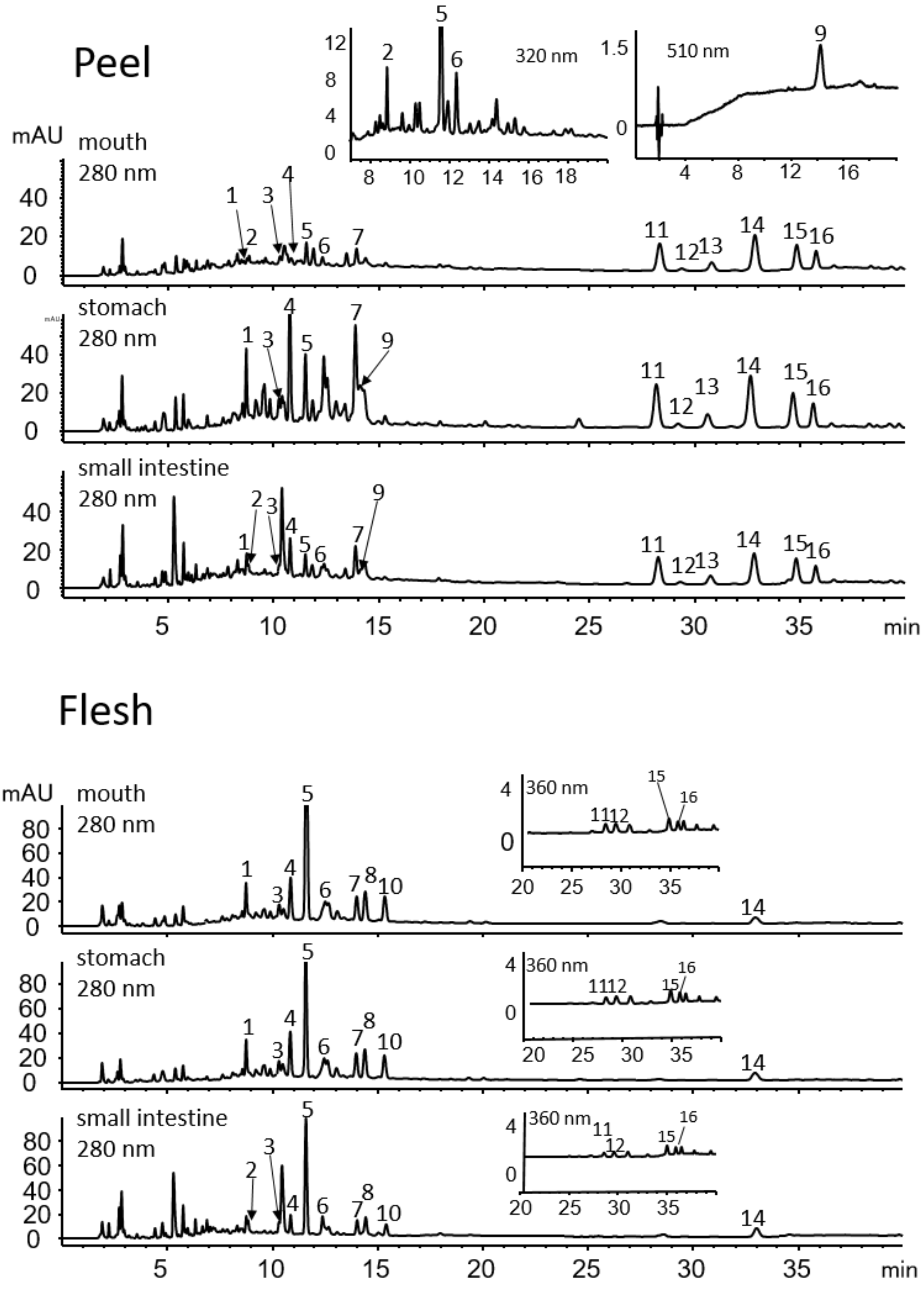

Figure S2. Chromatograms after the simulated digestion in the mouth, stomach and small intestine – apple peel and flesh. Peak identification: 1 – procyanidin B1, 2 – neochlorogenic acid, 3 – (+)-catechin, 4 – procyandin B2, 5 – chlorogenic acid, 6 – cryptochlorogenic acid, 7 – (-)-epicatechin, 8 – unknown phenolic acid, 9 – cyanidin-3-galactoside, 10 – *p*-coumaroylquinic acid, 11 – quercetin-3-galactoside, 12 – quercetin-3-glucoside, 13 – quercetin derivative, 14 – phloretin-2′-glucoside, 15 – quercetin-3-xyloside, 16 – quercetin-3-rhamnoside.
